# Y-Complex Proteins Show RNA-Dependent Binding Events at the Cell Membrane and Distinct Single-Molecule Dynamics

**DOI:** 10.3390/cells11060933

**Published:** 2022-03-09

**Authors:** Rebecca Hinrichs, Nadiia Pozhydaieva, Katharina Höfer, Peter L. Graumann

**Affiliations:** 1 SYNMIKRO, Zentrum für Synthetische Mikrobiologie, Philipps Universität Marburg, Karl-von-Frisch-Str. 14, 35043 Marburg, Germany; rebecca.hinrichs@synmikro.uni-marburg.de (R.H.); nadiia.pozhydaieva@synmikro.mpi-marburg.mpg.de (N.P.); katharina.hoefer@synmikro.mpi-marburg.mpg.de (K.H.); 2 Fachbereich Chemie, Philipps Universität Marburg, Hans-Meerwein-Straße 4, 35043 Marburg, Germany; 3 Max-Planck-Institut für Terrestrische Mikrobiologie, Karl-von-Frisch Straße 16, 35043 Marburg, Germany

**Keywords:** RNA degradation, riboswitch, *Bacillus subtilis*, Y-complex, RNase, single-molecule tracking

## Abstract

Bacteria are dependent on rapid alterations in gene expression. A prerequisite for rapid adaptations is efficient RNA turnover, with endonuclease RNase Y playing a crucial role in mRNA stability as well as in maturation. In *Bacillus subtilis*, RNase Y in turn interacts with the so-called “Y-complex” consisting of three proteins, which play important functions in sporulation, natural transformation and biofilm formation. It is thought that the Y-complex acts as an accessory factor in RNase Y regulation but might also have independent functions. Using single-molecule tracking, we show that all three Y-complex proteins exhibit three distinct mobilities, including movement through the cytosol and confined motion, predominantly at membrane-proximal sites but also within the cell center. A transcriptional arrest leads to a strong change in localization and dynamics of YmcA, YlbF and YaaT, supporting their involvement in global RNA degradation. However, Y-complex proteins show distinguishable protein dynamics, and the deletion of *yaaT* or *ylbF* shows a minor effect on the dynamics of YmcA. Cell fractionation reveals that YaaT displays a mixture of membrane association and presence in the cytosol, while YlbF and YmcA do not show direct membrane attachment. Taken together, our experiments reveal membrane-associated and membrane-independent activities of Y-complex proteins and a dynamic interplay between them with indirect membrane association of YmcA and YlbF via YaaT.

## 1. Introduction

Control of messenger RNA (mRNA) stability is a central part of gene regulation in all cells. Extending or shortening the lifetime of an mRNA has a profound effect on protein expression. Generally, most mRNAs have a short lifetime, and their instability is fundamental for the control of protein levels [1,2,3]. The processing and degradation of mRNAs are usually initiated by an endonucleolytic attack [4], so the most efficient way to regulate mRNA decay is to control the steps that initiate degradation.

In *Escherichia coli* and *Bacillus subtilis*, endonucleases RNase E and RNase Y, respectively, are known for a global effect on mRNA turnover, as well as for specific RNA processing [2,3,5]. For example, the RNase Y is able to produce endonucleolytic cleavages in the 5′-untranslated regions (UTRs) of genes to produce alternative transcript isoforms with shortened leader sequences from their precursor mRNAs [6,7]. Thus, RNase Y plays an important role in the regulation of *B. subtilis*. Especially for bacteria, the regulation of mRNA turnover allows cells to control gene expression at a post-transcriptional level and thus react quickly to changing growth conditions [4]. In *B. subtilis*, RNase Y is not essential, but deletion of the corresponding *rny* gene leads to severely retarded growth [8]. RNase Y influences the intracellular levels of most transcripts [9,10], and affects the turnover of riboswitches [5].

Two RNase Y-containing complexes have been proposed to exist in *B. subtilis:* a “degradosome” containing three other RNases, an RNA helicase as well as glycolytic enzymes [11], comparable to the RNase E-based degradosome in *Escherichia coli* [12,13,14]. In contrast to *E. coli*, the degradosome in *B. subtilis* could only be isolated with cross-linking agents [11]. Thus, the nature of the putative mRNA degradosome is still rather unclear. Recently, it was reported that the so-called Y-complex (YlbF, YmcA, YaaT) physically interacts with RNase Y, possibly setting up a second RNase Y-containing complex [15,16]. In addition to biofilm formation, competence and sporulation (here, the complex acts on the phosphorelay, although this is currently under debate), it was shown that the Y-complex plays a role global in mRNA stability and is necessary for the processing of the *cggR-gapA* transcript in *B. subtilis* [17]. Interestingly, RNase Y is also essential for this post-transcriptional regulation [11]. Thus, the complex is necessary for the efficient maturation of operon mRNAs and influences the frequency of distinct riboswitches [17]. Because of its crucial role in mRNA cleavage in vivo, Y-complex proteins have been proposed to act as accessory factors that regulate RNase Y activity [17]. An additional function for the Y-complex (apparently consisting of a 1:2:1 stoichiometry of YaaT/YmcA/YlbF) has been proposed based on its two [4Fe-4S]^2+^ clusters [18], and its FAD-binding capability, in that the complex may be involved in redox regulation.

Interestingly, a functional RNase Y-GFP fusion forms distinct foci at the cell membrane [19], and the dynamics of these foci have recently been shown to be influenced by Y-complex proteins, to a different extent depending on the individual proteins [20]. Of these, YmcA could be purified as a soluble protein, in contrast to YaaT and YlbF, such that the complex can only be obtained by co-expression of all three proteins. Thus, YaaT and YlbF appear to be insoluble, and interestingly, YaaT has also been shown to localize as foci at the cell membrane [17], similar to RNase Y.

In our work, we addressed the question of how Y-complex proteins can affect processes such as mRNA maturation, which we would expect to take place on the nucleoids, where mRNA is synthesized [21], unless non-matured mRNAs move from the nucleoids into the membrane-attached RNase Y foci. To obtain a spatial and temporal resolution as high as possible, we studied dynamics of the three members of the Y-complex during exponential growth using single-molecule tracking (SMT). We show that the localization and dynamics of all three proteins change during a transcriptional arrest, which suggests active participation of membrane structures in mRNA decay. We show that Y-complex proteins also arrest at sites in the cell away from the cell membrane, strongly suggesting that it also acts on the nucleoids, and not solely at the cell membrane. Our data considerably revise and refine our understanding of Y-complex dynamics in cells and reveal distinct biophysical properties of the three proteins.

## 2. Materials and Methods

### 2.1. Growth Conditions

Bacterial strains, plasmids and oligonucleotides used in this study are listed in Supplementary Table S1. LB (Lysogenic Broth) medium was used for the cultivation of *E. coli* strains. LB medium was also used for protein production, *B. subtilis* cultivation (overnight) and for solid agar plates. To study the used *B. subtilis* BG214 strain in the exponential phase, the culture was incubated at 30 °C and 200 rpm. For SMT, the culture was cultivated in S_750_ minimal medium (100 ml: 10 ml 10 × S_750_ salt solution [1 l ddH_2_O; pH 7.0: 104.7 g MOPS, 13.2 g of (NH_4_)_2_SO_4_, 6.8 g of KH_2_PO_4_, 12 g of KOH], 1 ml 100 × metal solution [100 ml: 20 ml of MgCl_2_ (1 M), 7 ml of CaCl_2_ (1 M), 0.5 ml of MnCl_2_ (1 M), 1 ml of ZnCl_2_ (0.1 M), 1 ml of FeCl_3_ (50 mM), 5 ml of Thiamine hydrochloride (2 mg/ml), 17 µl of HCl (2 M)], 2 ml 50% fructose (*w/v*), 1 ml of 10% L-glutamate (*w/v*), 40 µl of 1% Casamino acids (*w/v*) [22]. When needed, antibiotics were added in the following concentrations: ampicillin 100 µg/ml, chloramphenicol 5 µg/ml, kanamycin 30 µg/ml, rifampicin 25 µg/ml. 0.5% of xylose was added from a 50% sterile filtrated stock solution in ddH_2_O. For *B. subtilis* BG214, methionine (50 µg/ml) and tryptophan (50 µg/ml) were added to the medium.

### 2.2. Construction of Strains

To create mV fusions of *B. subtilis*, we used plasmid pSG1164-mV. Plasmid pSG1164-mV, which encodes the corresponding fluorophore mV, creates a single-crossover of the desired gene with the introduced plasmid sequence, thereby generating a C-terminal fusion of the sequence to the fluorophore at the original locus of the gene [23]. To insert a homologous region required for plasmid integration, at least 500 bp of the C-terminus of the desired gene was cloned by Gibson Assembly [24] into the vector next to the linker and mV sequence. The oligonucleotide primers used for Gibson assembly had a homologous overhang of at least 20 bp, as shown in Supplementary Table S1. The transformed plasmids were extracted using a kit (Sigma-Aldrich, Merck KGaA, Darmstadt, Germany). The deletion strains were based on *B. subtilis* BG214 containing YmcA-mV constructs, and were generated by the transformation of cells with chromosomal DNA from *B. subtilis* 168 Δ*yaaT::kan trpC2*, Δ*ylbF::kan trpC2*, or Δ*ymcA::ery trpC2*, obtained from the *Bacillus* Genetic Stock Center (Columbus, OH, USA) [25]. The chromosomal DNA was extracted using a kit (innuPREP Bacteria DNA Kit, Analytik Jena, Jena, Germany).

### 2.3. Protein Fractionation

In total, 50 ml of lysogenic broth supplied with 5 µg/ml of chloramphenicol were inoculated from a 2 ml overnight culture. Cells were grown to a mid-exponential phase (OD600 of 0.6) at 30 °C, and were harvested. The resulting pellet was resuspended in 1 ml lysis buffer F (50 mM EDTA, 100 mM NaCl, pH 7.5, and Protease Inhibitor Cocktail cOmplete™: EDTA-free Protease Inhibitor Cocktail, Roche, Basel, Switzerland). Cells were enzymatically lysed by incubation with 2.5 mg/ml lysozyme for 30 min at 37 °C. The subsequent protein fractionation of parts of the samples is described below. For the SDS-PAGE, SDS loading buffer was added and the samples were incubated for 1 h at room temperature. All samples were loaded using 16 µl thereof onto a 12% mini-PROTEAN TGX Stain-Free SDS gel (Bio-Rad Laboratories, Hercules, CA, USA). Prior to Western blotting, the total protein load of the gel was controlled via stain-free imaging using UV exposure.

For protein fractionation of the cell lysate, first, a 50 µl sample was withdrawn for SDS-PAGE analysis. The remaining cell lysate was transferred to a centrifugation tube with a volume capacity of 4 ml. Samples were centrifuged in 4 ml, Quick-Seal, g-Max Polypropylene tubes (Beckman Coulter, Brea, CA, USA) and were filled up with lysis buffer F to ¾ of the total tube volume (dilution about three-fold). We used an ultracentrifuge (Optima XPN 80, Beckman Coulter, Brea, CA, USA), and a 70 Ti fixed-angle rotor. After centrifugation at 100,000× *g* for 1 h in the ultra-centrifuge, a 50 µl sample of the cytosol-containing supernatant was taken. The remaining supernatant was discarded. The remaining pellet was resuspended in 1 ml lysis buffer. Again a 50 µl sample (which is about three-fold concentrated relative to the cytosolic fraction) was taken for SDS-PAGE analysis. Resulting fractions were termed “lysate”, and after ultra-centrifugation, the supernatant (cytosolic proteins) “cytosol” and the pellet-containing membrane proteins “membrane”. As a control, wild type strain BG214 was used.

### 2.4. Northern Blot Analysis

Total RNA isolated from *B. subtilis* was analyzed by Northern blotting with target-specific RNA riboprobes for *cggR* and *gapA* transcripts. Probes for detection of *cggR*, *gapA* and 5S rRNA transcripts were generated from DNA oligonucleotides listed in Supplementary Table S1. The partially complement primers pairs were converted to dsDNA by PCR reaction with 2.5 U of JumpStart^TM^ Taq DNA Polymerase (Sigma Aldrich, St. Louis, MO, USA). Following DNA was precipitated with isopropanol, the pellet was dissolved in 100 µl Millipore water. Northern blot probes were transcribed in 100 µl scale in the presence of 40 mM Tris-HCl pH 8.1, 1 mM spermidine, 22 mM MgCl_2_, 0.01% Triton-X-100, 10 mM DTT, 5% DMSO, 3 µM DNA template, 0.1 mg/ml T7 RNA polymerase (lab-prepared stock), 20 µCi α-^32^P-ATP (Hartmann Analytik GmbH, Braunschweig, Germany), 4 mM of CTP, GTP and UTP and 2 mM of ATP at 37 °C for 3.5 h. Afterwards, residual DNA template was removed by DNase I digest (0.02 U/µl, Roche, Basel, Switzerland) at 37 °C for 30 min. Samples were phenol/chloroform extracted, isopropanol precipitated and the pellet resuspended in 50 µl Millipore water.

For Northern blot analysis, RNA samples were mixed with 2× PAGE loading dye (1× TBE in formamide containing xylene cyanol and bromphenol blue). ^32^P-labelled HR RiboRuler Ladder (Thermo Fisher Scientific, Walthman, MA, USA) was prepared using 20 µl HR RiboRuler Ladder, 40 µCi of γ-^32^P-ATP (Hartmann Analytik GmbH, Braunschweig, Germany), 1× Buffer A (Thermo Fisher Scientific, Walthman, MA, USA) and 20 U PNK (Thermo Fisher Scientific, Walthman, MA, USA) at 37 °C for 1 h. Total RNA was fractionated by agarose gel electrophoresis (1.2% agarose, 0.6% formaldehyde *v/v*, 1× MOPS buffer, pH 7) at 110 V. Using a capillary transfer, RNA was transferred from the agarose gel to Whatman Nytran SuPerCharge nylon blotting membrane (GE Healthcare, Chicago, IL, USA) in 10× SSC buffer, pH 7 overnight. The membrane was UV cross-linked and prehybridized in 20 ml ROTI^®^ Hybri-Quick (Carl Roth, Karlsruhe, Germany) for 30 min at 45 °C. 10 µl of previously radiolabeled RNA riboprobes were added to the prehybridized membrane and incubated for 3 h at 45 °C. The blots were washed twice for 20 min with wash solution 1 (2× SSC, 0.1% SDS *w/v*) and twice for 20 min with wash solution 2 (0.1× SSC, 0.1% SDS *w/v*) at 45 °C. Results were visualized using storage phosphor screens (GE Healthcare, Chicago, IL, USA) and Typhoon^TM^ biomolecular imager (GE Healthcare, Chicago, IL, USA).

### 2.5. Western Blot

The samples of protein fractionation were separated by SDS-PAGE and visualized by Western blotting. The detection was performed with a primary polyclonal α GFP antibody (1:5000), and secondary antibody goat-anti-Rabbit-IgG, peroxidase-conjugated (1:100,000) (Sigma Aldrich, St. Louis, MO, USA).

### 2.6. Fluorescence Microscopy

Fluorescence microscopy was performed with S_750_ medium cultivated cells. *B. subtilis* cells were grown at 30 °C and 200 rpm until an exponential phase. For wide-field epifluorescence microscopy, a Zeiss Observer A1 microscope (Carl Zeiss, Oberkochen, Germany) with an oil immersion objective (100× magnification, 1.45 numerical aperture, alpha PlanFLUAR; Carl Zeiss, Oberkochen, Germany) was used. The images were recorded with a charge-coupled-device (CCD) camera (CoolSNAP EZ; Photometrics, AZ, USA) and an HXP 120 metal halide fluorescence illumination with intensity control. Sample preparation was performed using round coverslips (25 mm and covering 5 µl cell culture with a 1% agarose pad. The agarose pads were made with S_750_ medium by sandwiching 100 µl of the melted agarose between two smaller coverslips (12 mm). Images were processed using ImageJ [26].

### 2.7. Single-Molecule Tracking

The individual molecules were tracked using custom-made slim-field setup on an inverted fluorescence microscope (Nikon Eclipse Ti-E, Nikon Instruments Inc., Tokyo, Japan). An EMCCD camera (ImagEM X2 EM-CCD, Hamamatsu Photonics KK, Hamamatsu, Japan) was used to ensure high-resolution detection of the emission signal, resulting in a calculated resolution of the position of the molecule down to 20 nm. The central part of a 514 nm laser diode (max power 100 mW, TOPTICA Beam Smart, Toptica, Munich, Germany) was used with up to 20% of the intensity (about 160 W cm^−2^ in the image plane) to excite samples fused to mVenus (using a laser filter set with BrightLine 500/24, dichroic mirror 520 and BrightLine 542/27), by focusing the beam onto the back focal plane of the objective. A CFI Apochromat objective (TIRF 100× Oil, NA 1.49) was used in the setup. The videos were recorded with 3000 frames and 40 ms stream acquisition, using the camera program NIS-Elements. The software Oufti [27] was used to set the necessary cell meshes. Utrack [28] was used for signal detection and track generation, with a minimum track length of six steps selected. A strong fluorescence signal from the sample would lead to incorrectly connected tracks. To be sure that the motion is only analyzed at the single-molecule level, a bleaching curve of each movie was calculated in ImageJ. The bleaching curve can be modeled as an exponential decay, and a threshold on the slope of less than 10% is used as a cut off. On this basis, the first 500 to 1000 frames were cut out for all movies (dependent on signal intensity of the strains) to ensure imaging at a single-molecule level. All cells were incubated in S_750_ medium to the exponential phase at 30 °C and 200 rpm. The statistical data analysis was carried out according to already established methods [29,30].

## 3. Results

### 3.1. Localization of the Y-Complex in Live B. subtilis Cells

RNase Y has been shown to form discrete fluorescent foci at the cell membrane in exponentially growing cells [20], and similarly YaaT [17]. In order to obtain further knowledge on the role of the Y-complex in *B. subtilis*, we created C-terminal mVenus fusions (termed “mV” from here on) to each protein of the three proteins of the complex, which were integrated via single-crossover integration into the respective gene locus. To ensure the functionality of the Y-complex proteins with fusions, we performed sporulation assays and transformability tests. Both experiments showed that all fusions retained close to wild type-like function: cells carrying single fusions showed even slightly higher sporulation efficiency for YaaT and YmcA, and normal activity for YlbF (Figure S1A), and transformability was normal, except for the YmcA-mV fusion that showed slightly reduced activity compared with wild type cells devoid of any fusion (Figure S1B). As an additional control, we assayed for the processing of the *cggR-gapA* mRNA, which depends on the activity of RNase Y as well as that of the Y-complex proteins [17]. Figure S2 shows that while the *cggR-gapA* transcript fails to be cleaved in each strain lacking a component of the Y-complex, all the mVenus fusion strains are proficient in this activity, except for a minute accumulation of non-matured transcripts. Lastly, growth of fusion strains was very similar to that of cells not carrying any fusion construct (Figure S1C). These assays indicate that all proteins retained their functionality as protein fusions, and showed only a minimal reduction in functioning, as is often seen in fusion proteins.

**Figure 1:**
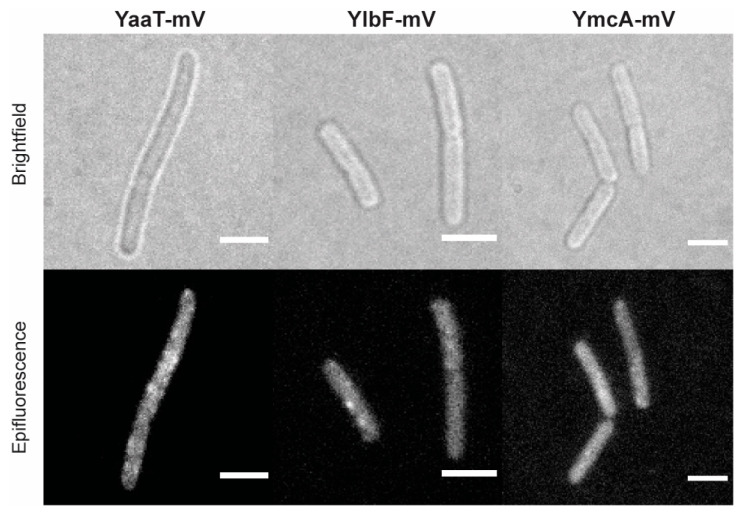
Localization of the Y-complex proteins expressed from native locus by epifluorescence in *Bacillus subtilis* (BG214). Scale bars 2 µm.

**Figure 2:**
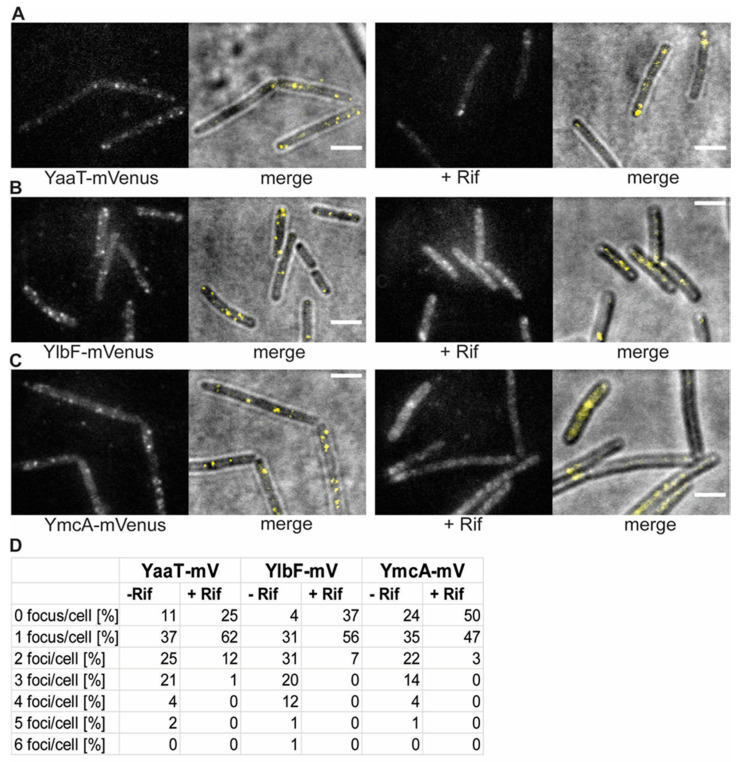
Changes in the localization patterns of Y-complex proteins during the exponential growth phase and in phase response to transcription arrest, by treatment with rifampicin for 30 min (“+ Rif”). (**A**–**C**): Localization by slim-field illumination. (**A**–**C**) Panels show the localization of the three different Y-complex proteins expressed from the native locus. Merge displays overlay of bright field and fluorescence. Scale bars 2 μm. (**D**): Percentage of the number of foci in cells with fluorescent signal before and after treatment with rifampicin.

Using epifluorescence (Figure 1) revealed that YaaT-mVenus forms fluorescent foci, mostly close to the cell membrane, similar to what was reported before for YaaT [31]. For YlbF-mV and YmcA-mV we also observed foci, but seemingly fewer per cell than for YaaT-mV. In order to obtain a better view on the spot-like assemblies of Y-complex proteins, we used slim-field microscopy, where the central part of a laser diode is focused on the back focal plane of the objective, resulting in high light intensity and slightly divergent illumination of the image plane. This technique allows to track single molecules at a time scale of milliseconds; adding up images from a whole movie of 3000 frames blurs out diffusing molecules and reveals the presence of molecules at similar positions, forming discrete foci. Using this approach yielded more clearly defined foci (Figure 2) and revealed the existence of many foci in a majority of cells, mostly but not always close to the cell membrane.

Counting the number of foci in about 200 cells for each strain, from a biological triplicate, showed that few cells did not contain any foci, and most cells showed between one and two visible foci (Figure 2D). The number of foci contains an error based on subjective counting of fluorescent signals. Interestingly, when cells were incubated with rifampicin, inhibiting the activity of RNA polymerase, the number of foci per cell dropped, and the number of cells lacking foci increased (Figure 2). Thus, the components of the Y-complex behave similarly towards rifampicin treatment, corresponding to the finding of complex formation by the three proteins [18]. It has been shown that RNase Y still forms foci at the cell membrane after inhibition of transcription [20], but that RNase Y relocalizes from the membrane towards to cytosol [32]. Because there is a dispute over whether SMT data on RNase Y are valid, we will perform additional control experiments before further proceeding with a comparison between RNase Y and Y-complex protein dynamics in a future study.

### 3.2. Dynamics of Y-Complex Proteins at a Single-Molecule Level Reveals Rapid Binding and Unbinding Events

In order to better quantify changes in molecule dynamics of Y-complex proteins in response to transcription inhibition, we employed SMT microscopy [33,34]. With this method, we were able to detect the localization of Y-complex fusion proteins with a higher spatiotemporal sensitivity than with epifluorescence or TIRF microscopy. Briefly, the beam of a 514 nm laser diode was widened 20-fold, and the central part was focused on the back focal plane of the 100× A = 1.49 objective. Images were captured in stream acquisition using an EM-CCD camera. With SMT we are able to visualize events of molecules resting at a defined subcellular site with a precision of 40 nm and less [35], and can detect freely diffusive molecules in living cells [21]. The generated fusion proteins were tracked using 40 ms stream acquisition, because at this integration time, we obtained the highest number of tracks. After initial bleaching of most molecules, single-molecule tracks are captured for a total of 3000 frames (120 s). Cell meshes were determined using the software Oufti [27] and trajectories were determined by u-track [28]. Only consecutive tracks of five steps and longer were used for evaluation. Final analysis was performed using SMTracker 2.0, a custom-made graphical user interphase program [32]. Data for each represented experiment were collected from three independent biological replicates.

**Figure 3:**
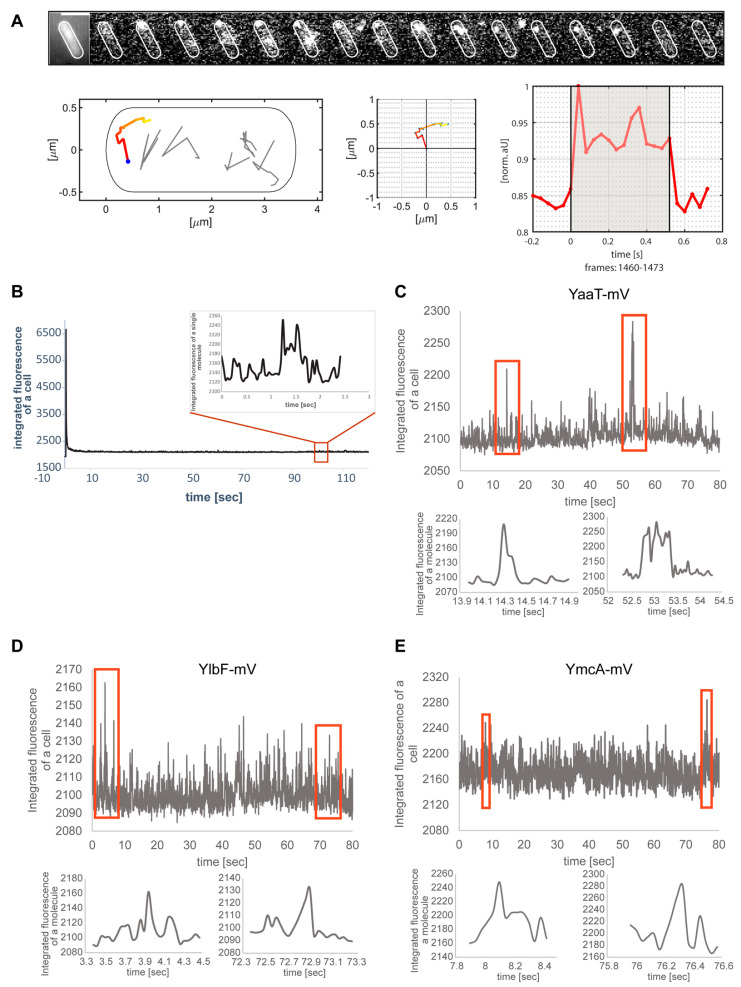
Single-molecule/particle tracking of Y-complex proteins. (**A**) Exemplary track of YaaT-mV. Montage shows images captured by 40 ms stream acquisition. Middle panels show motion of the focus within 2D, and fluorescence in arbitrary units. (**B**) Bleaching curve of the movie from panel A, inset shows track from panel A. (**C**) Examples of fluorescence for two or three molecules adding to a YaaT assembly within single frames, (**D**,**E**) track events for YlbF-mV or for YmcA-mV.

Figure 3A shows an example of a single-molecule/particle track, in this case travelling along the cell pole. The 2D distance travelled can be seen in the standardized cell, and in the coordinate system (Figure 3A). Fluorescence of the focus is bleached in one step in the final frame, which can be seen in the fluorescence plot. The latter also reveals that the intensity of the fluorescent focus roughly doubled at least once (more likely twice) during the lifetime of the particle trajectory, suggesting that at least one molecule attached to the structure containing YaaT within the observation time. Figure 3B shows the bleaching curve, from which the trajectory was taken. Interestingly, events of binding of new molecules to existing YaaT-mV assemblies were frequently observed for YaaT (Figure 3C, note that formally, maturation of newly attached YaaT-mV molecules is observed, which is stochastic). Average background fluorescence was 2100 a.u., and that is single YaaT-mV molecules about 2150 a.u. Interestingly, events of 100 or 150 a.u. increase during a single frame was observed (Figure 3C), showing that two or three molecules can associate with YaaT-mV molecules within 40 ms intervals. These observations suggest exchange rates of 20 ms and less for YaaT from the Y-complex, faster than e.g., 40 ms reported for exchange of MotB from the rotating flagellum [36]. Expecting that there are fewer than 22 copies of YaaT within a mobile Y-complex (as opposed to MotB within the flagellar machinery), the exchange rate of YaaT is much faster than that of MotB. Similar events of at least two molecules adding to existing structures within one frame could be observed for YmcA (Figure 3E), but more rarely than for YaaT, and very rarely for YlbF (Figure 3D), indicating different exchange rates of YaaT, YlbF and Y+mcA from the Y-complex.

### 3.3. Single-Molecule Dynamics Can Be Best Explained by Assuming Three Distinct Populations with Distinct Mobilities

**Figure 4:**
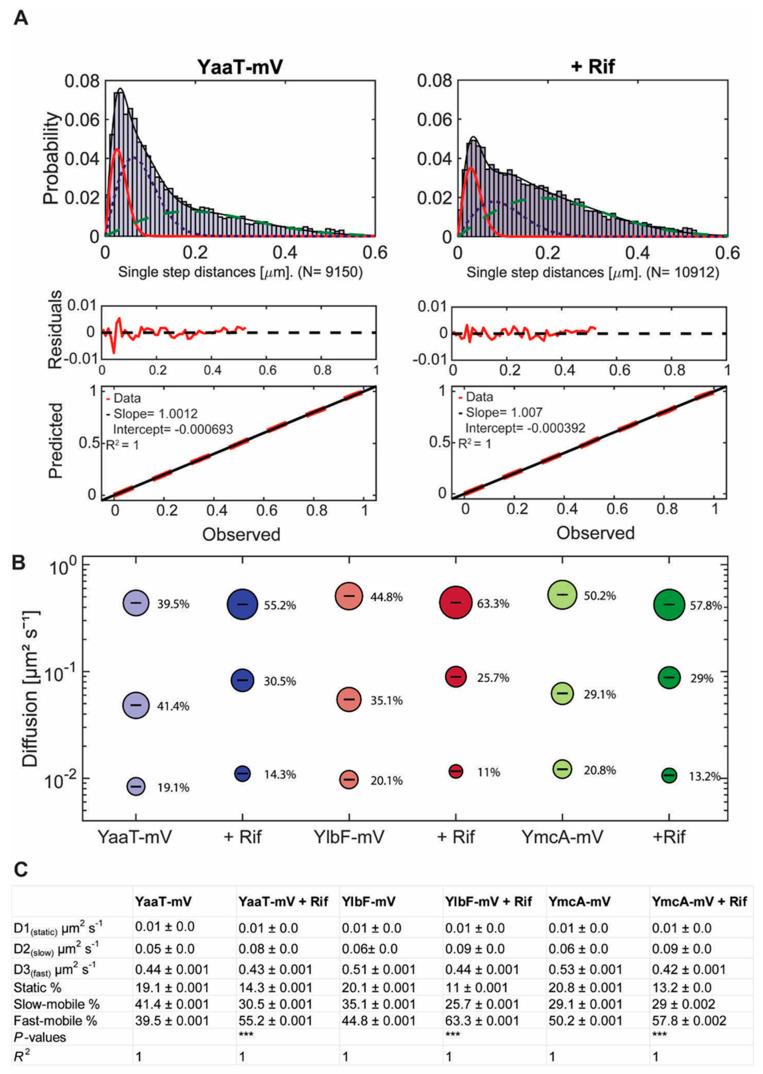
Squared displacement (SQD) analysis of Y-complex-mV fusion proteins under different conditions in cells growing in mid-exponential phase. (**A**) Three Rayleigh distributions were used to fit the observed data and to calculate diffusion coefficients for individual diffusive populations (YaaT-mV: N (number of trajectories) = 6141, YaaT-mV + Rifampicin: N = 14,339). Values show diffusion coefficients and their fraction sizes calculated by a nonlinear least-squared fitting method by the native MATLAB function. The frequency of the diffusion constants (probability density) was plotted against the specific diffusion coefficient of each track in a histogram. R^2^ value is annotated for each histogram. Diffusion of Y-complex proteins can be described best with a three-population fit representing a static (red solid line), slow-diffusive (blue dotted line) and one fast-diffusive (green dashed line) population. The lower two panels show quantile–quantile plots, where the difference between measured data and modeled data (indicated by straight red dotted line) is shown by the blue curve (see Figure S3 for the corresponding JD analyses for two populations). (**B**) Bubble plot shows diffusion constants [µm^2^ s^−1^] of mVenus fusions and fraction size of populations [%]. (**C**) Diffusion constants and percentages of static, slow-mobile and fast-mobile molecule fractions. The errors correspond to the 95% confidence intervals given by the MATLAB function “confint”, which uses for its calculation the values that result from the fit. Low confidence intervals are due to the large data sets. *p*-value: Symbol *** indicates for *p*-values lower than 0.001. The *R*^2^ value of each fit is annotated.

Jump distance is defined as the Euclidean distance between consecutive detections. A shift of movement of molecules towards larger displacement after addition of rifampicin is clearly visible in Figure 4A, showing that YaaT molecules become more dynamic after rifampicin treatment. Squared displacement (SQD) analysis was employed to quantify obtained displacements, which uses the cumulative probability distribution of the quadratic shifts to estimate the diffusion constants, and to determine if the distribution of displacements can be explained by a single population of molecules, or if more populations have to be assumed in order to explain the data. The number of populations is determined via a non-linear least-square fitting procedure [32]. When the distribution of jump distances was fitted with two Rayleigh distributions, deviations were well visible (Figure S3A), and using three distributions explained the observed data much better (Figure S3B). Residuals that did not agree with the predicted fit (based on Brownian motion) were smaller for three than for two distributions (Figure S3B, compare with A), and quantile–quantile (Q–Q) plots showed that the observed data (red line Figure S3) depicted a higher deviation from the modeled data (black dotted line, Figure S3) for two than for three Rayleigh fits. These analyses strongly suggest that three populations with distinct diffusion coefficients exist for YaaT in vivo, which was also found for YmcA and YlbF. Figure 4B visualizes the data shown in Figure 4C: the size of the bubbles corresponds to the relative size of the population, the height along the Y-axis to the diffusion constant. Note that errors reported are fitting errors (see material and methods for details of error determination), all data are from three independent biological replicates. It is apparent that diffusion constants of the slowest population (from here on called the “static” fraction) are very close to each other, with D = 0.01 µm^2^ s^−1^ for YaaT-mV, D = 0.01 µm^2^ s^−1^ for YlbF-mV, and D = 0.01 µm^2^ s^−1^ for YmcA-mV, suggesting that about 20% of the proteins form one complex of a very large size, based on minimal diffusion.

The medium-mobile populations of all three proteins had similar diffusion constants as well, varying between 0.05 and 0.06 µm^2^ s^−1^. This value is similar to that reported for translating 70S ribosomes [37,38], indicating that this population of Y-complex proteins is also part of a complex of a considerable size. Because it is unlikely that the Y-complex is associated with active translation, we propose that this fraction might be Y-complex proteins bound to mRNA, directly or indirectly (see further below). The high mobile fractions are of about 0.5 µm^2^ s^−1^, quite low for molecules we would expect to be freely diffusive. To test if using 40 ms stream acquisition might lead to an underestimation of the diffusion constant of freely diffusing molecules, whose motion may be blurred out at this acquisition speed, we tracked the three proteins using 20 ms integration time. Diffusion constants of the fast-mobile fractions rose to 1.1 ± 0.01 µm^2^ s^−1^ for YaaT-mV, 0.97 ± 0.02 µm^2^ s^−1^ for YlbF-mV, and 1.3 ± 0.02 µm^2^ s^−1^ for YmcA-mV, which is in the range of diffusion constants obtained for other freely diffusive proteins in *B. subtilis* [33,39]. Taken together, we interpret these findings as support of the presence of three different mobilities for Y-complex proteins, with an underestimation of the diffusion constants of the medium and fast mobile fractions at 40 ms, which has to be kept in mind, but does not generally compromise further results obtained.

### 3.4. Inhibition of Transcription Leads to Large Changes in the Dynamics and of the Location of Confined Motion

We wished to analyze the behavior of Y-complex proteins during the depletion of mRNA at the single-molecule level, to investigate changes in dynamics in the absence of substrate. For this, we incubated the cell culture for 30 min with rifampicin, after it had reached exponential phase. Rather than using 200 µg/ml as in standard protocols, we treated cells with 25 µg/ml, because we found that even with 100 µg/ml treatment, some cells started to show cell lysis after 30 min. We chose a reduced concentration of rifampicin to be able to investigate cells that are still alive, with the caveat that possibly overall transcription was not completely blocked, but considerably reduced. SQD analysis after treatment with rifampicin revealed a shift in population sizes towards a higher diffusion constant. The diffusion constant of the static population of YaaT-mV remained similar at 0.01 µm^2^ s^−^^1^ and the size of this population decreased from 19% to 14%. The medium-mobile population strongly increased almost two-fold in mobility, from 0.05 to 0.08 µm^2^ s^−1^, but decreased in size, while the fast-mobile population showed an increase from 40% to 55% (Figure 4C). For comparison, we also tracked glycolytic enzyme PfkA, suggested to be a part of the RNA degradosome [11]. Although obtained tracks could be well explained by the existence of two populations, we chose three populations for comparison with Y-complex proteins. Figure S5B,C show a mild decrease in the static fraction of PfkA following rifampicin treatment, and less than two-fold increase in the diffusion constant of the putative medium mobility fraction. Thus, even though some PfkA molecules may be involved in RNA degradation via the degradosome [11,32], it shows much less pronounced increases in mobility during RNA depletion. This control experiment shows that the significant shift of the diffusion constant of the medium-mobile fraction of YaaT-mV after transcription arrest, and the strong reduction in the static population, reflects a strong involvement of YaaT in mRNA binding, directly or indirectly, in accordance with its participation in global mRNA stability [5,9]. For YlbF-mV and YmcA-mV, we found a similar strong reduction in the static fraction, and a shift in diffusion constant of the medium-mobile fraction, with a concurrent increase in the fast mobile/freely diffusing population.

**Figure 5:**
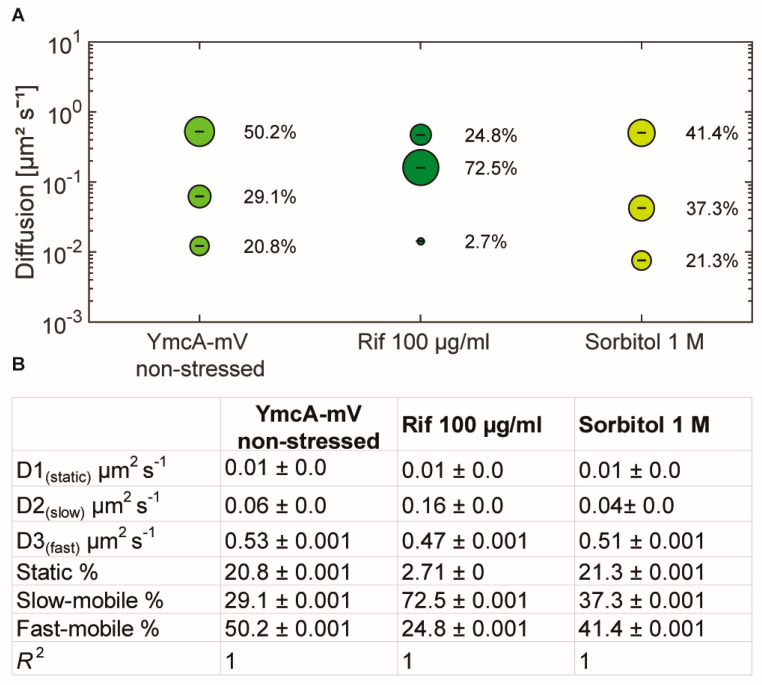
Squared displacement analyses of YmcA single-molecule dynamics. (**A**) bubble plot showing fractions sizes and their corresponding diffusion constants, “Rif” indicates cells treated with 100 µg/ml of rifampicin for 15 min, “Sorbitol” indicates cells incubated with 1 M sorbitol for 30 min. (**B**) Data from SQD analyses.

As an important control, we tested if the increase in overall mobility of molecules of the Y-complex observed after rifampicin treatment could be an effect on the viscosity within the cells, as addition of rifampicin has been shown to decrease the mobility of a very large protein complex in *E. coli* cells [40]. Using YmcA as a representative of the complex, we treated cells with an increased dose of rifampicin (100 µg/ml) for 15 min (still trying to avoid too many dying cells). We observed an even stronger decrease in static fraction, to below 3% (compared with 13% using the lower concentration), and an even stronger increase in the diffusion constant of the medium-mobile fraction, to 0.16 µm^2^ s^−1^, as opposed to 0.06 µm^2^ s^−1^ in non-stressed cells or 0.09 µm^2^ s^−1^ in cells after the lower rifampicin dose (Figure 5). While population sizes changed in favor of the medium-mobile population (likely reflecting a loss of sharpness in the distinction between medium and freely mobile molecules), no drastic effect was seen after osmotic upshift by the addition of 1 M sorbitol to the medium, which only slightly increased the size of the medium-mobile fraction and lowered its diffusion constant. Of note, it has recently been shown that translating ribosome (polysome)-induced crowding at sites surrounding the nucleoids (i.e., predominantly at the cell poles) is accompanied by lowered diffusion of large enzymes, but not of smaller proteins [41]. This is in agreement with our observation of the shift in mobility of the medium-mobile YmcA molecules, but not of the freely diffusive ones (Figure 5), supporting the view that increased crowding via osmotic stress affects the mobility of larger protein complexes, but not of small proteins such as YmcA. These experiments support the idea that the medium-mobile population of Y-complex proteins is associated with mRNA, either directly or indirectly.

**Figure 6:**
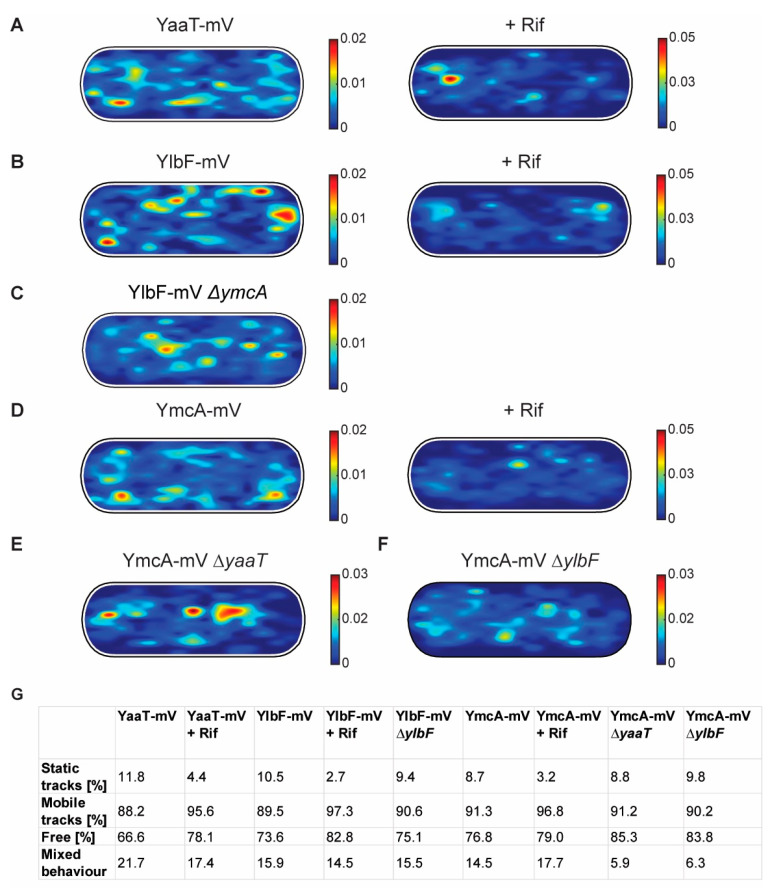
Confinement maps of Y-complex proteins with and without rifampicin, and of YmcA-mV in the absence of YaaT or YlbF. (**A**–**F**) Plots of heat map of confined tracks, projected into a standardized *B. subtilis* cell. Constructs as indicated above the panels. (**G**): Overview of ratios between static and mobile tracks.

We next projected all tracks from the three biological replicates into an average size cell of 3 × 1 µm size (“confinement heat map”, *B. subtilis* cells are on average 0.75 µm wide and 2 to 4 µm long). Tracks were sorted into those that stay within a radius of 120 nm, determined as three times our localization error (deduced from the intercept of MSD graphs), for at least 5 consecutive steps (confined motion), and into those that show large displacements, indicative of free diffusion. Figure 6 shows that during exponential growth, confined motion was largely biased towards the cell membrane/the cell periphery, for all three proteins. However, especially for YaaT and YlbF, confined motion also occurred away from the cell membrane (Figure 6A,B). Of note, confined motion of Y-complex proteins was different from that of the—mostly polarly localized—ribosomes showing confined motion, as represented by L1-mV (Figure S4) [42], indicating that Y-complex proteins are not associated with translating ribosomes.

In response to RNA depletion, all three Y-complex proteins showed a lower degree of confined motion, and absence of membrane-oriented confinement (Figure 6A,B,D). About 12% of YaaT-mV tracks were static (i.e., showing purely confined motion) during growth, and only about 4% following rifampicin treatment (Figure 6G). A similar effect was found for YlbF and YmcA (Figure 6G). To further quantify the effect of mRNA depletion of Y-complex dynamics, mobile tracks were sorted into those that are non-confined for the entire length of the track (“free”) and those that show a period of confined motion, representing molecules that undergo a transition between free diffusion and confined motion (“mixed behavior”). Transition events accounted for about 22% of YaaT molecules, and 16 or 15% for YlbF and YmcA, revealing a somewhat higher degree of binding and unbinding events to larger structures/complexes for YaaT. After rifampicin treatment, the number of transitions was lower for YaaT, but remained similar for YlbF, and was only mildly increased for YmcA, showing slightly different changes in the dynamics of the three proteins.

Figure S5A shows all tracks, confined, freely diffusing, and transitions, in a standardized cell for YaaT. Rifampicin treatment leads to strong relocalization of all kinds of tracks away from the cell membrane towards the cell center for YaaT, but not for PfkA. Additionally, mobility of PfkA-mV only moderately increased after rifampicin treatment (Figure S5B,C), in agreement with an earlier report [32], and different from the strong changes seen for Y-complex proteins (Figure 4B,C). Please note that molecules showing confined motion will largely overlap, but are not necessarily identical with those that show static motion in SQD analyses.

Relocalization of molecules away from the periphery towards the cell center following treatment with rifampicin was also found in heat maps, in which all tracks were projected into a standardized cell (Figure S6). These experiments support findings made with wide field fluorescence microscopy that peripheral accumulation of molecules, which corresponds to confined motion, depends on transcription (Figure 1) and thus on the availability of mRNA as a substrate for the Y-complex.

### 3.5. Deletion of YaaT or of YlbF Has a Moderate Influence on the Dynamics of YmcA-mV

We wished to investigate if the absence of Y-complex proteins has a profound effect on the motion of another protein from the complex. We therefore investigated the localization and dynamics of the most mobile fusion, of YmcA-mV, after deletion of *yaaT* or *ylbF*. For this purpose we transformed the fusion strain YmcA-mV with DNA from *yaaT* or *ylbF* deletion strains (Table S1). In addition to the transformation ability, we were able to test the function of the strains with a sporulation assay (Figure S1A). YmcA-mV/Δ*yaaT* cells showed a reduced sporulation capacity of 24.6% compared to the wild type (set to 100%) or 57.5% for the YmcA-mV Δ*ylbF* strain.

Interestingly, the deletion of *yaaT* led to a strong shift of confined YmcA-mV tracks away from the periphery towards the cell center (Figure 6E). These experiments suggest that YaaT may be a membrane anchor for the Y-complex. The deletion of *ylbF* also led to noticeable but less pronounced changes in the localization of YmcA-mV tracks (Figure 6F).

**Figure 7:**
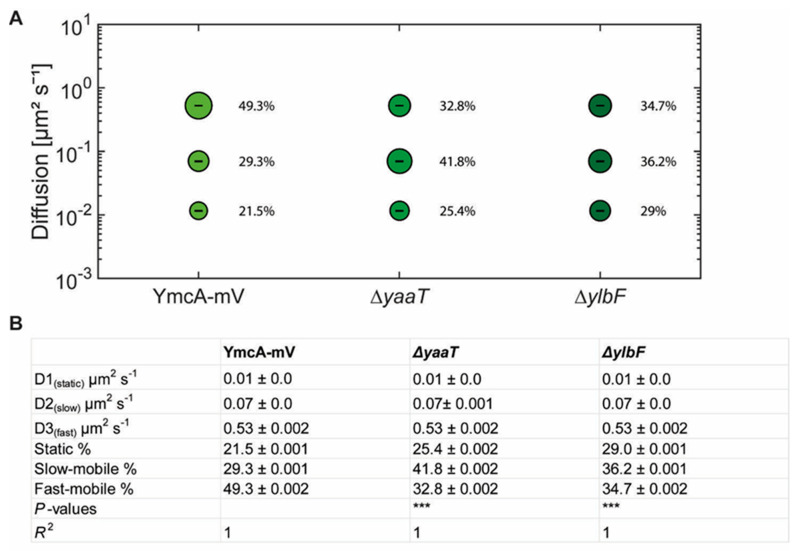
(**A**) Bubble blots showing squared displacement analysis of YmcA-mV under different conditions in cells grown to mid-exponential phase. Bubble blot shows diffusion constants [µm^2^ s^−1^] of YmcA-mV, YmcA-mV Δ*yaaT* and YmcA-mV Δ*ylbF* cells and comparison of fast-mobile, slow-mobile and static fraction sizes [%]. (**B**) Diffusion constants and percentages of static, slow-mobile and fast-mobile molecule fractions. *p-*value: The symbol *** represent *p*-values lower than or 0.001.

In SQD analyses, we observed considerable changes in YmcA dynamics, but surprisingly, in a counterintuitive manner (Figure 7). For the purpose of this analysis, a common average diffusion constant was determined for populations for all three strains, such that changes in dynamics are represented by changes in population sizes only. Although we would have expected YmcA to lose binding to the putative complex with RNase Y, YmcA-mV became more static in the absence of YaaT, and more so in cells lacking YlbF (Figure 7A). Also, in both Δ*yaaT* or Δ*ylbF* cells, the medium-mobile fraction of YmcA-mV increased considerably from about 30% to 36% or over 40% (Figure 7B), and the fast-mobile fraction decreased. Whatever the nature of the complex containing medium-mobile YmcA molecules (and likewise medium-mobile YaaT and YlbF molecules), its steady state-composition during exponential growth depends on the presence of all Y-complex proteins. On the other hand, lack of YaaT or YlbF does not abolish the ability of YmcA to become statically positioned, likely representing its binding to the very slow-moving RNA degradosome or at least a complex containing RNase Y, based on its dependence of the availability of (m)RNA. To determine expression levels, we performed Western blot analyses of YmcA-mV, using anti GFP antiserum, in wild type and in *yaaT* or *ylbF* deletion strains. Figure S7A shows that YmcA-mV was about two-fold more abundant in both mutant strains than in wild type cells. Possibly, absence of YaaT or of YlbF reduces overall RNA degradation, leading to increased YmcA levels. For higher-than-normal levels of YmcA, an increase in freely diffusive molecules would have been expected, due to a possible lack of binding sites on partner proteins, but the opposite was observed in the SQD analyses. Also, higher levels of YmcA cannot explain a complete relocation of confined tracks from the cell membrane towards the cell center. Poised by this finding, we tested for YlbF abundance in the absence of YmcA, and found a strong, roughly eight-fold reduction in YlbF levels (Figure S7B). This unexpected observation suggests that YmcA might affect the stability of YlbF in cells. Remaining YlbF-mV lost predominantly membrane-associated confined motion in Δ*ymcA* cells, and was mostly found within the cytosol at the center of cells (Figure 6C). Thus, lack of Y-complex components affects intracellular protein levels as well as protein mobility of other components.

These experiments suggest that the Y-complex is a highly dynamic entity, where on- and off-binding events are affected by the presence of all three proteins, rather than a complete loss of complex formation.

### 3.6. Y-Complex Proteins Show Different Degrees of Association with the Membrane

**Figure 8:**
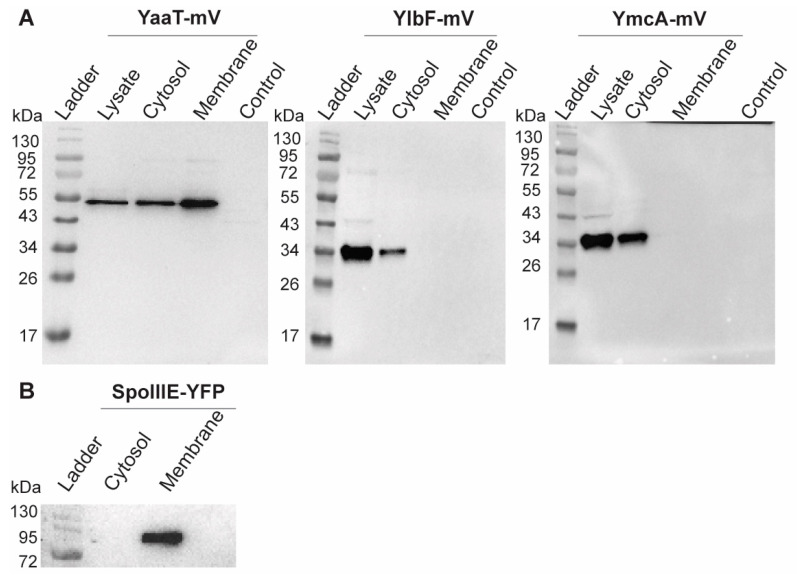
Western blots showing cell fractionation of (**A**) Y-complex proteins or (**B**) of SpoIIIE as mV or YFP fusions, respectively. Total cell extracts from exponentially growing cultures (LB) were used. The YaaT-mV fusion (57.9 kDa), YlbF-mV (43.7 kDa), YmcA (42.9 kDa) contains the mVenus polypeptide (26.9 kDa). Note that YlbF runs erroneously low, possibly because samples have not been heated before loading to the gel, which yields clearer bands. All strains were detected via GFP-antiserum. The fractionation was carried out by ultracentrifugation (100,000× *g*). The displayed fractions shown are full lysate, supernatant (Cytosol) and pellet (Membrane). “Control” BG214 wild type cells without mVenus fusion.

Heat maps generated from SMT experiments suggested that YmcA loses membrane association in the absence of YaaT (Figure 6E). We therefore performed cell fractionation and subsequent Western blot analyses of exponentially growing cells. As a control for successful fractionation, the integral membrane protein SpoIIIE-YFP (86.96 kDa + 27 kDa mVenus) was analyzed, which was exclusively present within the membrane fraction (Figure 8B) [43]. YaaT was found in the membrane fraction and in the cytosolic fraction (Figure 8A). Figure S8 shows a fractionation in which the amount of membrane proteins is loaded such that cytosol plus membrane proteins equal the amount of protein from the lysate, showing that YaaT is present in each fraction to roughly equal parts. This finding is in agreement with the difficulty of obtaining soluble YaaT protein [18], but also agrees with our observation of diffusive as well as confined motion of YaaT within the cytosol. Different from YaaT, YlbF and YmcA were only present within the cytosolic fraction (Figure 8A). Please note that the faint bands seen in the YmcA Western blot are cross-reactions that run at different heights than YmcA-mVenus. These data show that YlbF and YmcA have different physical properties than YaaT in vivo, and suggest that YaaT serves as an anchor for the Y-complex at the cell membrane. We cannot distinguish at present if YaaT has membrane association via strong binding to RNase Y, or via an intrinsic membrane-binding activity.

## 4. Discussion

RNA turnover is an important way of controlling gene expression, an essential trait for bacteria to rapidly adapt to changing environmental conditions. In *E. coli* and many other bacterial species, RNase E plays a key role in global mRNA decay, and does so in conjunction with several other proteins, including other RNases, an RNA helicase, and a glycolytic enzyme, enolase [44]. Interestingly, bacterial species from many different phyla possess RNase Y, which appears to play an analogous function as RNase E, in initiating RNA decay through endonucleolytic attacks [9]. RNase Y also appears to be part of an RNA degradosome [11,45]. Intriguingly, RNase Y has also been implicated in being part of a second complex, the so-called Y-complex (or RicAFT, for regulatory iron-sulfur containing complex subunits A, F and T): YaaT, YlbF and YmcA form a soluble complex [18], and are involved in RNA turnover as well as in specific RNA maturation events that also include RNase Y [10,16,17,46]. However, many features of the Y-complex are unclear. While YlbF can be purified separately, YaaT cannot, and only becomes soluble when all three proteins are present. RNase Y membrane assemblies are affected in their mobility by Y-complex proteins in *B. subtilis* [20], but in an unknown manner. It is also curious to note that RNase Y and Y-complex play a role in gene regulation occurring on the nucleoids, e.g., in riboswitch activities [17]. There are precedents of membrane-localized transcription factors [47,48], so obviously, Y-complex regulated operons might translocate to the cell membrane, analogous to the *lac* operon moving to the cell membrane when transcription of the lactose permease is induced [49], whose transcription is thought to be directly coupled to its membrane insertion.

We addressed some of these intriguing questions by studying the dynamics of all three proteins of the Y-complex at a single-molecule level. We reasoned that binding to different protein complexes or RNA substrates might lead to Y-complex proteins being present in different states of mobility, which would change upon loss of interaction partners or substrate. We found that three fractions with distinct average diffusion constants could well account for all molecule dynamics. They could make sense because YmcA, YlbF and YaaT might be associated with differently sized protein/RNA complexes.

RNA endonuclease activity for RNase Y toward operon mRNA maturation and degradation requires the Y-complex [17]. We show that very similar to RNase Y, YaaT, YlbF and YmcA also show considerable events of confined motion within the cell, indicating that they participate in RNase Y activity taking place on the nucleoids. Further key findings of our work are: (a) Y-complex proteins not only show confined motion close to the cell membrane (which likely correspond to the foci observed by epifluorescence microscopy), but also within the cytosol. Because confined motion can most easily be explained by binding to a larger structure showing little movement, it is likely that YmcA, YlbF and YaaT arrest at sites of transcription where mRNA substrates for RNase Y and the Y-complex are synthesized (e.g., riboswitches); (b) we also find freely diffusive tracks within the cytosol. This is in complete agreement with cell fractionation experiments showing that about half of YaaT molecules are strongly attached to the cell membrane, and 50% are cytosolic, while YlbF and YmcA are only found in the cytosolic fraction. These findings imply that YaaT serves as a membrane anchor for the other two Y-complex proteins. This idea is supported by our finding that the absence of YaaT in the cell leads to a shift in confined motion for YlbF away from the cell membrane, towards the cell center, and vice versa. Interestingly, YmcA levels in the cell about double in the absence of YaaT or of YlbF, while YlbF is present in a highly reduced amount in Δ*ymcA* cells. The most straightforward explanations for these observations are a reduction in *YmcA* mRNA turnover in the absence of YaaT or YlbF, while YlbF may require YmcA for protection against proteolysis. However, several alternative scenarios could be the case too. Cell fractionation also shows that the components of the Y-complex have different biophysical properties with regard to membrane association, and that the complex might be highly dynamic in terms of its composition. This is underlined by our finding that the overall dynamics of YlbF do not change drastically in the absence of YaaT or YlbF, which we would have expected would the proteins form a defined stoichiometric complex that falls apart in the absence of components.

Our findings also reveal that (d) inhibition of transcription leads to a loss of confined motion at the cell membrane, and thus to a decrease in membrane recruitment for all three Y-complex proteins. This strongly suggests that Y-complex membrane assemblies are active structures forming in a substrate-dependent manner. It will be of importance to study whether the foci correspond to RNA degradosome assemblies, or to independent structures.

SQD analysis of obtained tracks strongly suggested that all three Y-complex proteins are present in at least three distinct populations showing different mobilities. We observed a fraction of about 20% of molecules that showed extremely low mobility, lower than that described for translating ribosomes. This fraction could be part of the RNA degradosome, also containing RNase Y and several other RNases, an RNA helicase and two glycolytic enzymes. Such a large complex bound to possible multiple RNAs could indeed show observed low mobility. A second, largest fraction of molecules showed mobility similar to that of translating ribosomes. We speculate that this population could be a cytosolic complex bound to mRNA, which is then transported to the membrane-localized RNA degradosome. The fast-moving fraction of molecules likely corresponds to freely diffusing YaaT, YmcA and YlbF proteins, or a small, soluble subcomplex of e.g., YmcA and YlbF; these proteins showed most similar single-molecule dynamics and solubility, and were quite small compared with YaaT, which showed membrane association for a large portion of molecules. Our finding that the diffusion constant of the medium-mobile fraction became much faster, while the fast-moving fraction remained largely unaffected after rifampicin treatment suggests that the fast-mobile fraction indeed consists of freely diffusive Y-complex molecules, while the medium-mobile fraction is apparently involved in RNA binding.

The differential effects of loss of individual Y-complex proteins suggests that in vivo, there could well be different associative forms of the Y-complex, which might explain the different effects of the individual mutants observed: the Y-complex has been reported to accelerate the phosphorylation of the transcription factor Spo0A, contribute to genetic competence, sporulation and biofilm formation, and to be essential for the correct maturation of multiple protein coding and riboswitch RNAs in *B. subtilis* [16,17,18]. Our analyses suggest that Y-complex proteins associate in a highly dynamic manner, and do not form a single defined complex. For example, the SMC chromosome segregation complex shows complete loss of the static DNA-bound fraction upon loss of one of its three subunits, and the diffusion constant of ScpA subunits increases drastically when the ScpB protein, which ScpA forms a subcomplex with, is absent from cells [34,50]. Such clear-cut changes in molecule dynamics were not observed with Y-complex proteins in cells lacking a component of the putative complex.

Pioneering work by the Dubnau group has shown that the Y-complex can only be purified by co-expression of all three proteins, and has a complex structure [46]. We found that localization of YmcA-mV and single-molecule dynamics are affected by the loss of YaaT as well as YlbF. However, we still found YmcA being mobile within cells, indicating that in vivo, at least YmcA can remain soluble in the absence of its complex partners. Collectively, our data support the idea that the Y-complex can exist in different forms in *B. subtlilis*, and reveal that the Y-complex is intimately associated with RNA dynamics at the cell membrane and within the cytosol. It remains an intriguing question how the four proteins can associate so dynamically, assuming such different functions.

## Data Availability

All relevant data are shown withion this study. Upon reasonable request, raw data for single molecule tracking movies can be obtained from the corresponding authors.

## Supplementary Materials

The following are available online at https://www.mdpi.com/article/10.3390/cells11060933/s1, Figure S1: Test for functionality of the Y-complex protein fusions in Bacillus subtilis cells, Figure S2: Maturation of the gapA operon mRNA in *B. subtilis*, Figure S3: Jump-distance histograms to compare a double or triple population Rayleigh fit for YaaT-mV in *B. subtilis* cells, Figure S4: Confinement maps of Y-complex proteins, Figure S5: Confinement maps of YaaT-mV and of PfkA-mV during exponential growth and after rifampicin treatment, Figure S6: Heat maps of single-molecule localization of Y-complex proteins, Figure S7: Western Blot analyses of YmcA-mV expression levels, Figure S8: Fractionation of YaaT-mV. Table S1: Strains and primers used in this study.
