# Supplementary material for: Y-Complex Proteins Show RNA-Dependent Binding Events at the Cell Membrane and Distinct Single-Molecule Dynamics
Source: Cells. 2022 Mar 9;11(6):933. doi: 10.3390/cells11060933 (PMC8945944; doi:10.3390/cells11060933)
Supplement: Supplementary file 1 [file cells-11-00933-s001.zip › cells-1580000-supplementary.pdf]

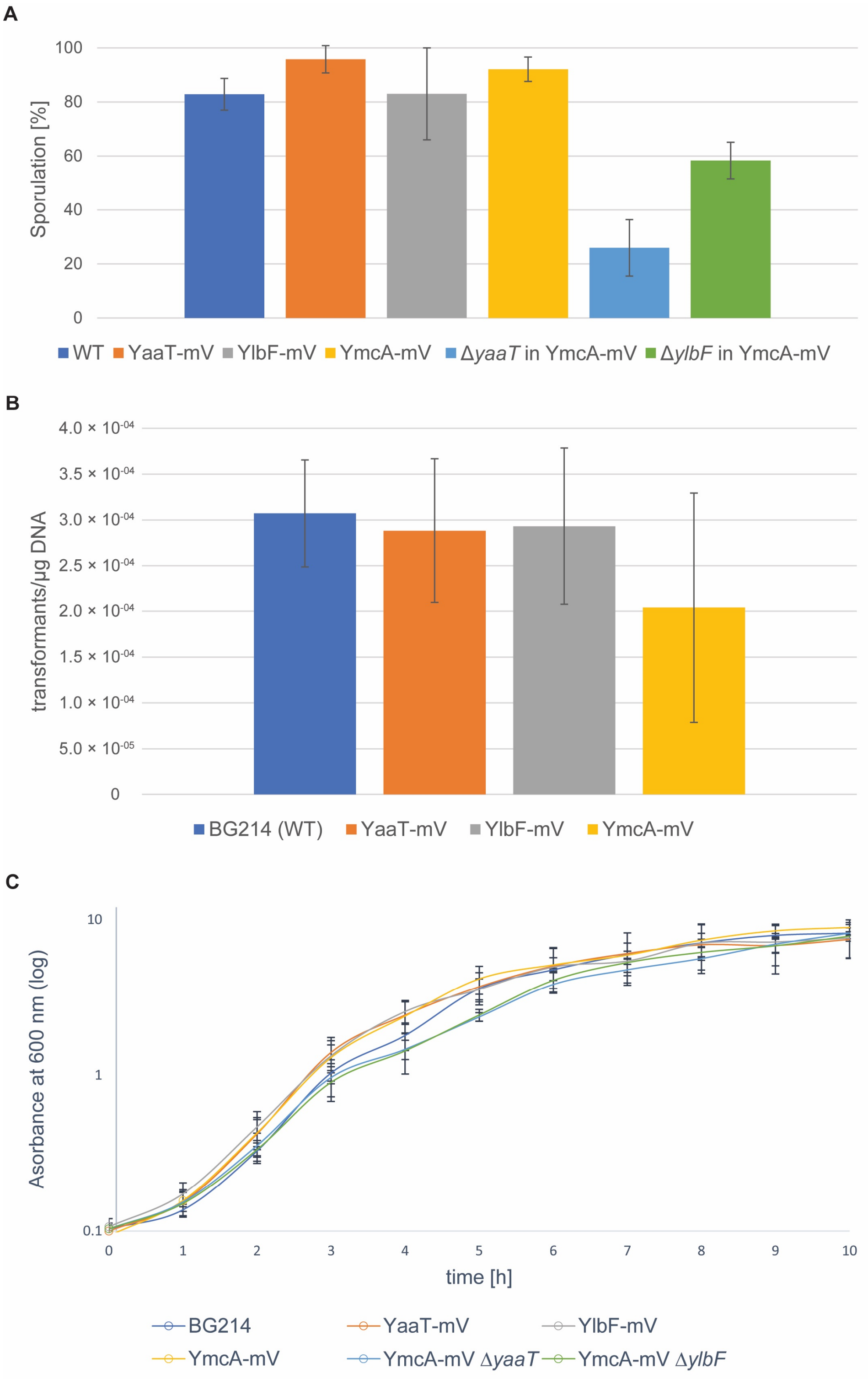

Figure S1. Test for functionality of the Y-complex protein fusions in *Bacillus subtilis* cells. **A**) Bar plot with error bars shows the percentage of sporulation after incubation in DSM-culture medium in comparison to the wild type. The wild-type represents a sporulation of 82.5%. The mVenus fusions showed no compromise in the function of the sporulation (YaaT-mV 95.5%, YlbF-mV 81.7%, YmcA-mV 92.1%). The deletions of *yaaT* and *ylbF* in YmcA-mV clarify a reduced function of the sporulation. *P*-value: The symbols *, ** and *** indicate *P*-values lower than 0.1, 0.05 and 0.01, respectively, n.s. statistically not significant. **B**) Transformation assay. Bar plot with error bars shows the percentage of transformants/µg DNA in comparison to the wild-type strain. Assays were performed in technical triplicates and in biological duplicates. Error bars indicate

standard deviation. **C**) Growth curves for strains containing the following strains: BG214 (darkblue), YaaT-V (orange), YlbF-mV (grey), YmcA-mV (yellow), YmcA-mV Δ*yaaT* (light blue), YmcA-mV Δ*ylbF* (green). Measurements were performed in 300 ml flasks with a volume of 50 ml.

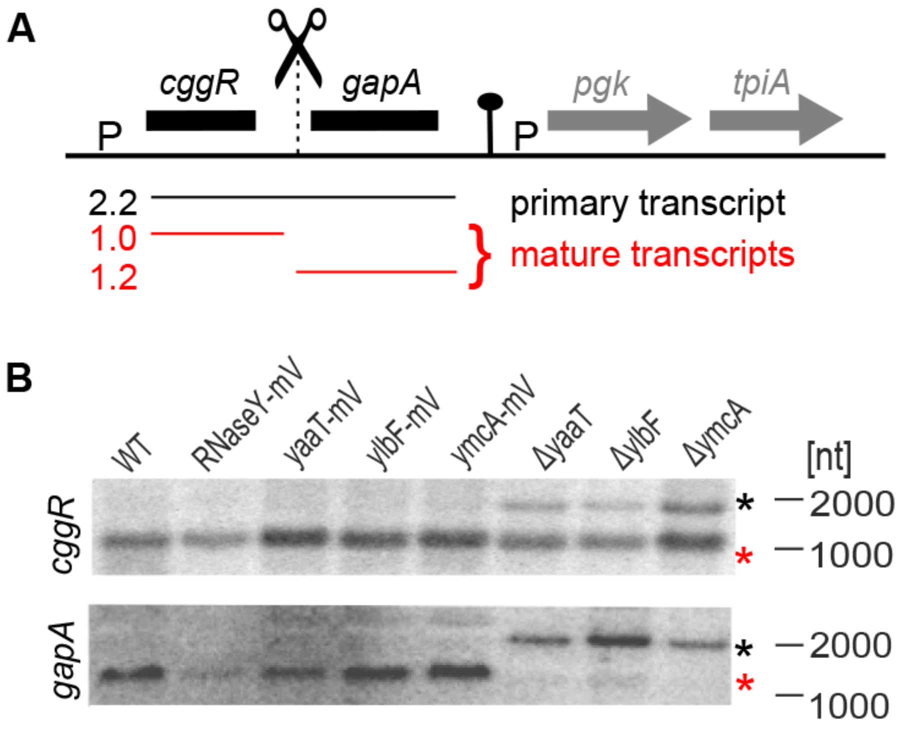

Figure S2. Maturation of the *gapA* operon mRNA in *B. subtilis*. **A**) Schematic illustration of the *gapA* operon primary transcripts processed by RNase Y. The maturation of the RNA results in two transcripts *cggR* (1.0 kb) and *gapA* (1.2 kb). **B**) Analysis of the processing of *cggR* and *gapA* transcripts in different *B. subtilis* strains. Processed *cggR* or *gapA* transcripts are visualized by Northern blot. Primary (black asterisk) and mature transcripts (red asterisk) of the *gapA* operon are detected by specific riboprobes.

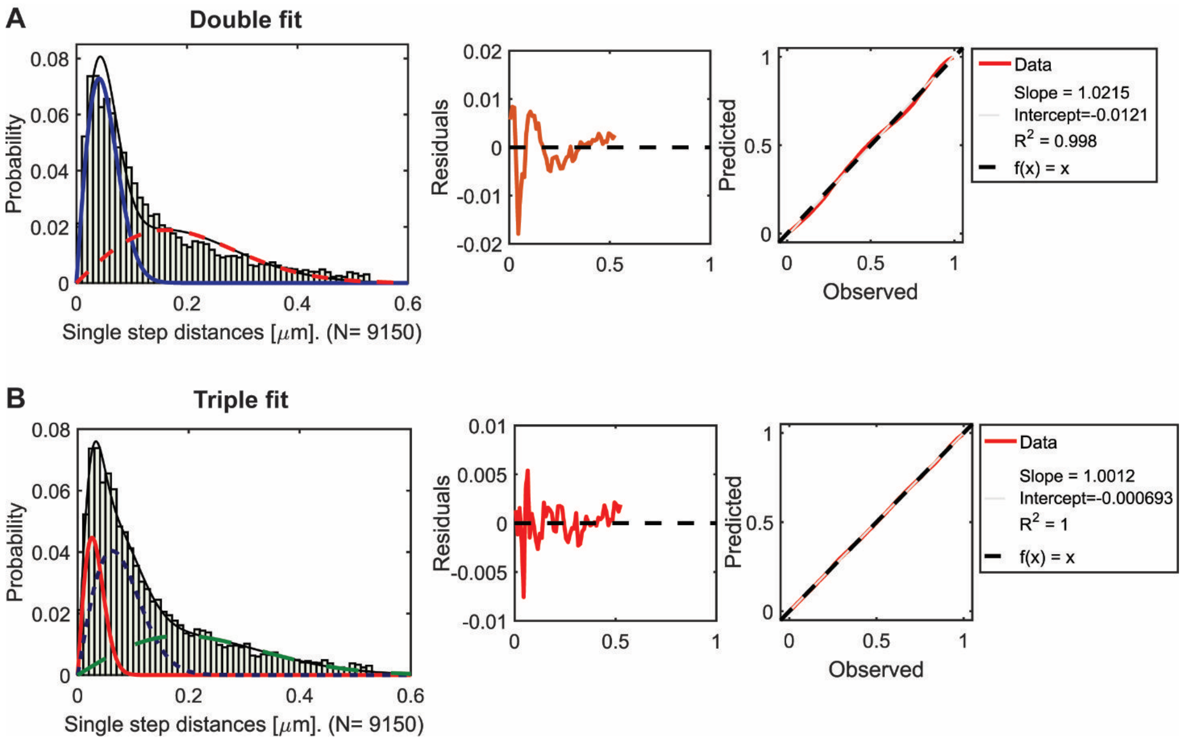

Figure S3. Jump-distance histograms to compare a double (**A**) or triple population (**B**) Rayleigh fit for YaaT-mV in *B. subtilis* cells. Histograms of jump-distance values overlaid with each individual group model, static (green solid line in (**A**), red solid line in (**B**)), slow-diffusive (blue dotted line in (**B**), and diffusive (green dotted or dashed line)), and their sum (black solid line). Lower panels are quantile-quantile plots, showing the difference (blue curve) between measured data and modelled data (represented by the dashed red line), and the resulting R^2 determined the goodness-of-fit.

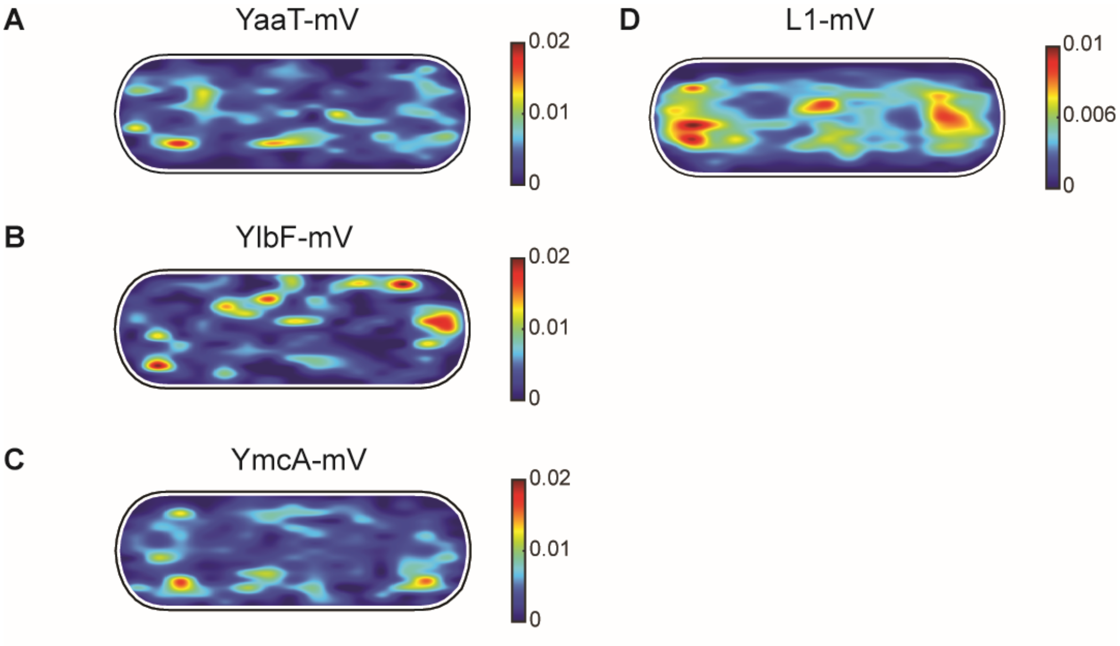

Figure S4. Confinement maps of Y-complex proteins compared with that of L1-mV representing ribosomes, projected into standardized (medium-size) *B. subtilis* cells. Strains in panels **A-D** as indicated above the maps.

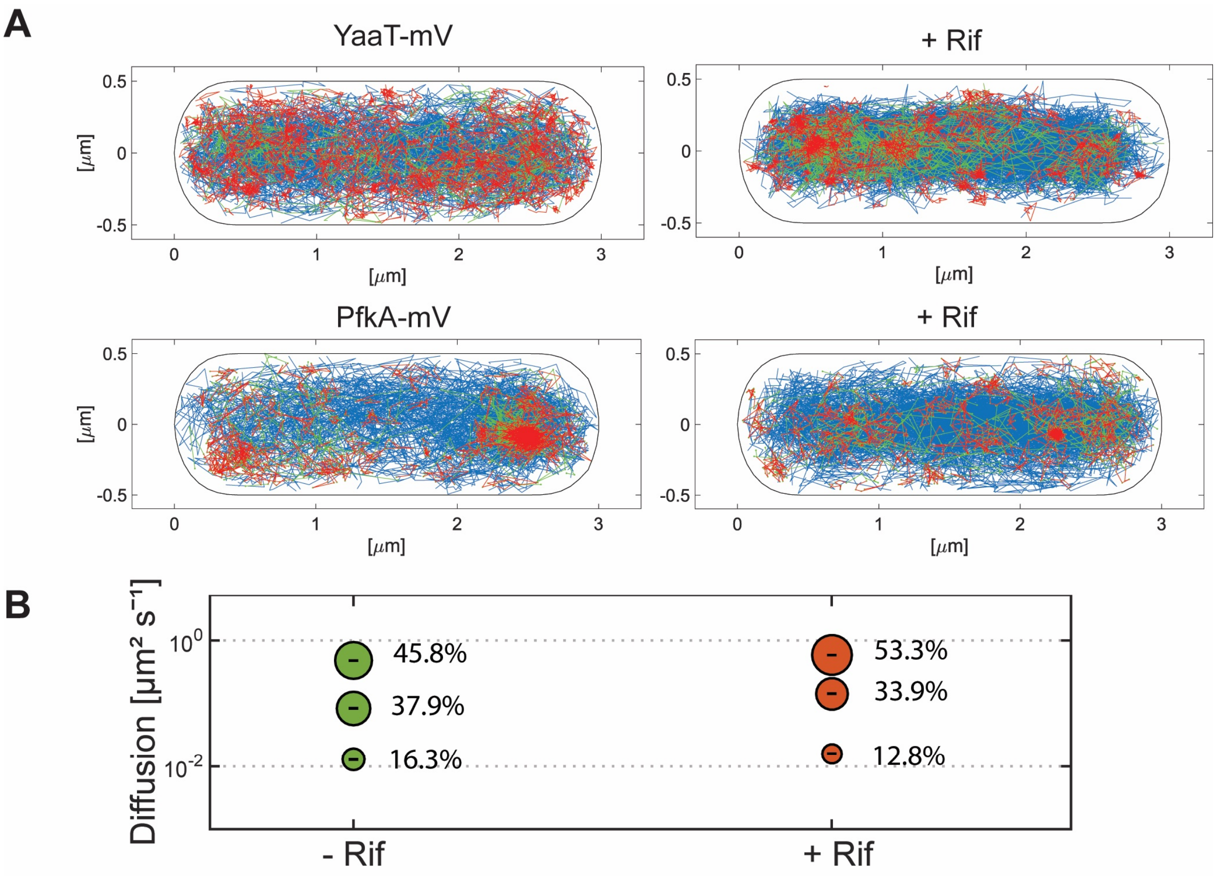

C

	PfkA-mV (-Rif)	PfkA-mV (+Rif)
$D1_{(static)}$ $\mu m^2/s^{-1}$	0.01 ± 0.0	0.02 ± 0.0
$D2_{(slow)}$ $\mu m^2/s^{-1}$	0.08 ± 0.001	0.14± 0.001
$D3_{(fast)}$ $\mu m^2/s^{-1}$	0.49 ± 0.002	0.58 ± 0.002
Static %	16.3 ± 0.002	12.8 ± 0.001
Slow-mobile %	37.9 ± 0.002	33.9± 0.001
Fast-mobile %	45.8 ± 0.002	53.3 ± 0.001
R^2	0.99997	0.99998

Figure S5. A) Confinement maps of YaaT-mV and of PfkA-mV during exponential growth and after rifampicin treatment. Plots of tracks classified into confined (red, staying within a radius of 120 nm for at least 6 steps), mobile (blue) and tracks performing transitions (green), in a standardized *B. subtilis* cell. **B**) Bubble blots show diffusion constants of PfkA-mV without and with treatment of Rifampicin and fractions sizes for fast-mobile, slow-mobile and static molecules. **C**) Diffusion constants and percentages of static, slow-mobile and fast-mobile molecule fractions. Values were fitted using non-linear least-square fitting, R^2 values for each condition are stated.

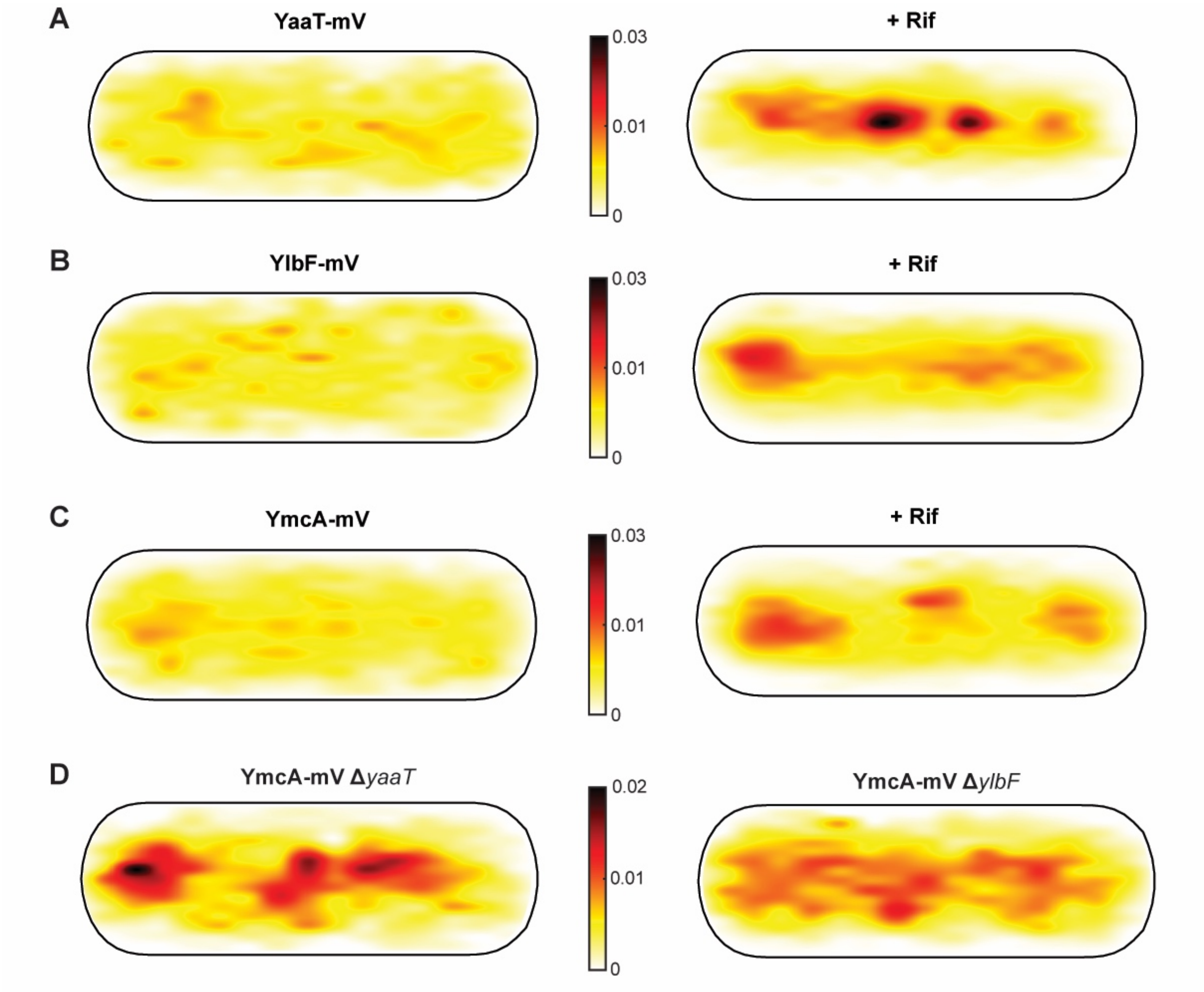

Figure S6. Heat maps of single-molecule localization of Y-complex proteins in a standardized *B. subtilis* cell. The distribution of tracks is indicated by a color shift from yellow (low probability) to black (highest probability). **A**) A YaaT-mV fusion localized throughout the cells. "+Rif" YaaT-mV fusion after treatment with rifampicin for 30 min, revealing a relocation away from the membrane towards the cell center. **B**) YlbF-mV as in panel A, **C**) YmcA-mV fusion as in panel A. **D**) YmcA-mV fusion in the absence of YaaT or of YlbF.

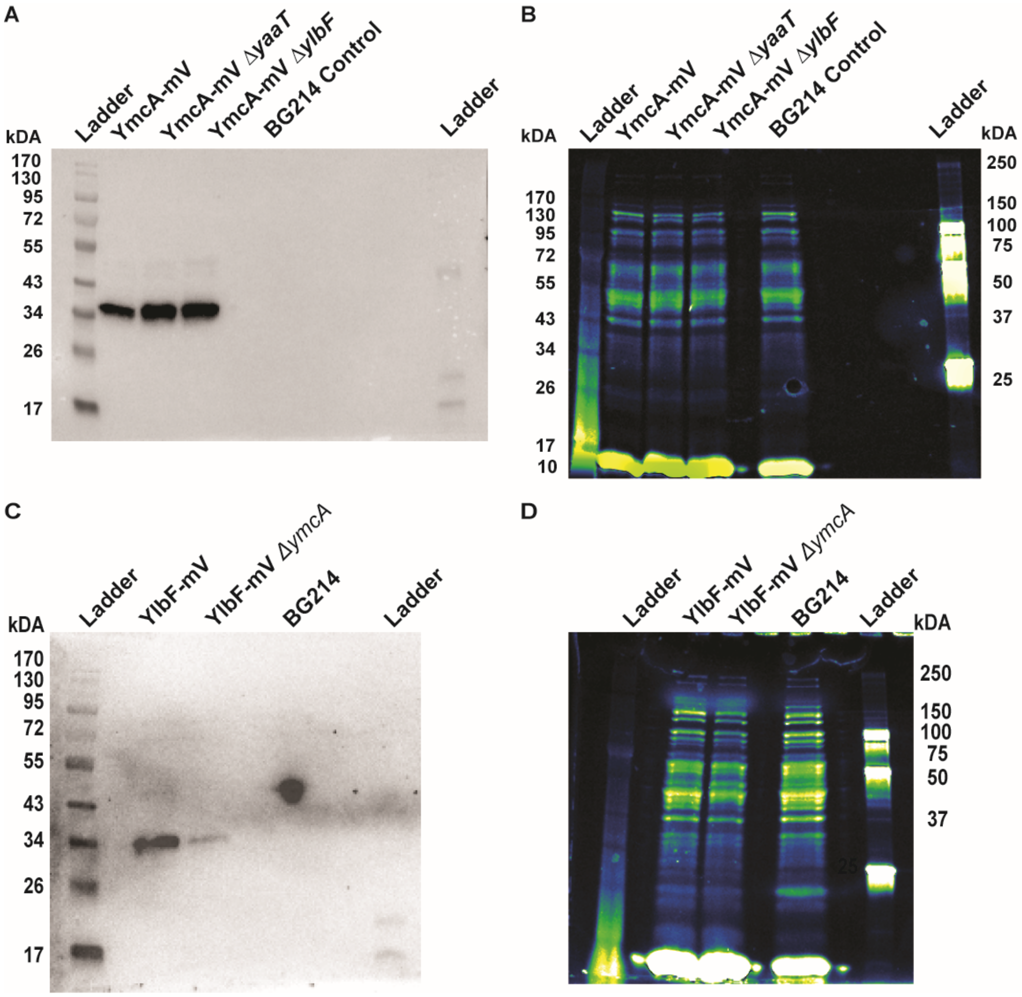

Figure S7. Western Blot analyses of YmcA-mV expression levels in the absence of YaaT or of YlbF. **A**) Western blot analysis of YmcA-mV and the deletion strains Δ*yaaT* and Δ*ylbF*. **C**) Western blot analysis of YlbF-mV in comparison to the deletion Δ*ymcA.* The wild type strain BG214 was used as a control. All strains were detected via GFP-tag-antiserum. **B**, **D**) Prior to western blotting, the total protein load of the gel was controlled by stain-free imaging, using Bio-Rad PROTEAN TGX Stain-Free SDS gels that allow for UV-activated quantitative imaging using UV exposure, before the gels are blotted onto a membrane.

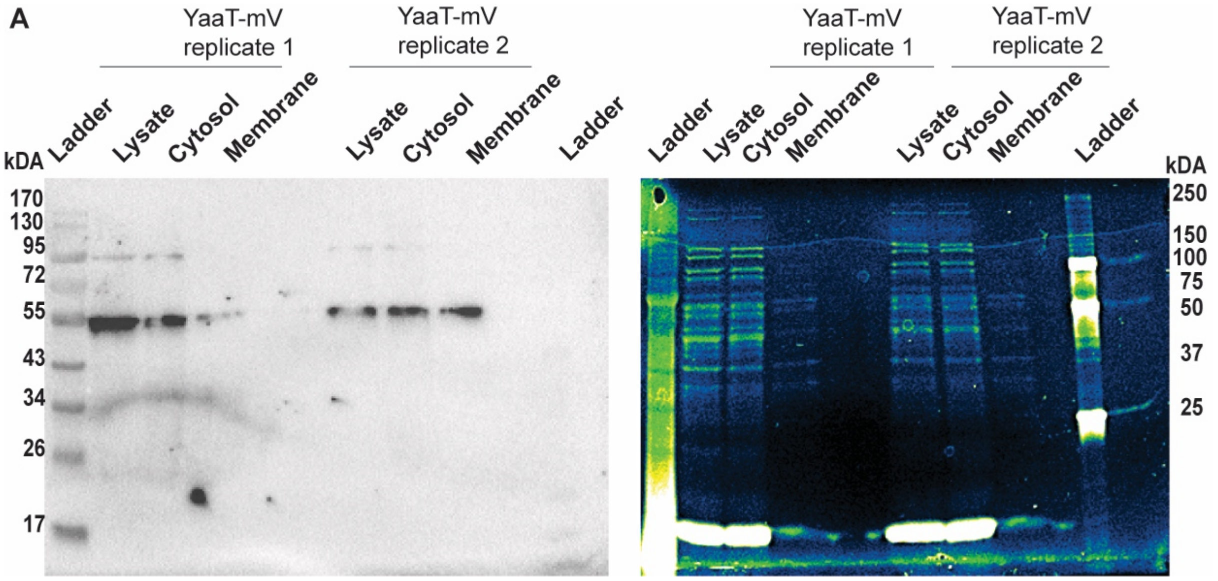

Figure S8. Fractionation of YaaT-mV. **A**) Western blot analysis of YaaT-mV fractionation assays, shown are two independent experiments (revealing some degree of variability). Samples were loaded such that the three-fold dilution of the cytosolic fraction relative to the lysate due to centrifugation was equalized, and the pellet containing membrane fractions was resuspended in the same volume as the cytosolic fraction. **B**) Loading control, protein bands were visualized using UV excitation of Bio-Rad PROTEAN TGX Stain-Free SDS gels.

Table S1 **Strains and primers used in this study**

**Strain or Plasmids**	**Relevant features**	**Reference or source**
B. subtilis		
BG214	Wild type	
PG3741	BKK14990 Δ*yaaT*::*kan trpC2*	[25]
PG3740	BKK00320 Δ*ylbF*::*kan trpC2*	[25]
PG3742	BKK17020 Δ*ymcA*::*kan trpC2*	[25]
PG3811	*yaaT-mVenus*^{cmR}	This study
PG3812	*ylbF-mVenus*^{cmR}	This study
PG3813	*ymcA-mVenus*^{cmR}	This study
PG3838	Δ*yaaT*::*kan trpC2 ymcA*-mVenus^{cmR}	This study
PG3839	Δ*ylbF*::*kan trpC2 ymcA-mVenus*^{cmR}	This study
PG4270	Δ*ymcA*::*erm trpC2 ymcA-mVenus*^{cmR}	This study
E. coli		
DH5α	*sup*E44 Δ*lac*U169 φ80d*lac*ZΔM15 *hsd*R171 *rec*A1 *end*A1 *gyr*A96 *thi*-1 *rel*A1	New England Biolabs (NEB)
PG3730	DH5α pSG1164-mVenus, expression vector, Amp^{R}, Cm^{R}	[23]
PG3852	DH5α pSG1164 *yaaT*-mVenus, expression Vektor, Amp^{R}, Cm^{R}	This study
PG3853	DH5α pSG1164 *ylbF*-mVenus, expression Vektor, Amp^{R}, Cm^{R}	This study
PG3854	DH5α pSG1164 *ymcA*-mVenus, expression Vektor, Amp^{R}, Cm^{R}	This study
**Primer**	**Sequence 5´→ 3´**	
PG3811 fw	CCTAGGATGGGTACCGAATTCCGTTTGATCGCAATAAAGT	
PG3811 rv	AGGCCAGATAGGCCGGGCCCATCTGTGGTTTGTGCGG	
PG3812 fw	CTAGGATGGGTACCGAATTCATGTATGCGACGATGGAATC	
PG3812 rv	AGGCCAGATAGGCCGGGCCCGGACACTTT ACATCCGC	
PG3813 fw	CTAGGATGGGTACCGAATTCGAAAATCCAGCAAGCGAAAA	

PG3813 rv	AGGCCAGATAGGCCGGGCCCGAGAGAACAGCTGTTAT
**Sequencing primer**	
mVenus/yfp rev	TGCGCTCCTGGACG
kan^R fw	CGCTCTACTCAAAAAAAGAC
kan^R rv	CTGTAAAGGCACCGTGTTTA
**Northern Blot**	
cggR_fw	TAATACGACTCACTATAGGCAATAATATCGGGGATGGCGTCTATGT CATC CAGCTGCATT
cggR_rev	CGGACGGCGAAGTGGTTCACAAAGTGCATTCTGTCGGAATGCAGC TGGATGACATAGACG
gapA_fw	TAATACGACTCACTATAGGCTGCAAGGTCAACAACGCGGTTAGAGT AGCCGCTTTCGTTA
gapA_rev	GGCAGCATGGTAAAAGTAATCTCTTGGTACGATAACGAAAGCGGCT ACTCTAACCGCG
